# Metabolic Alterations in Cancer Cells and the Emerging Role of Oncometabolites as Drivers of Neoplastic Change

**DOI:** 10.3390/antiox7010016

**Published:** 2018-01-17

**Authors:** Zhengqiu Zhou, Elochukwu Ibekwe, Yevgen Chornenkyy

**Affiliations:** 1 College of Medicine, University of Kentucky, 800 Rose St., Lexington, KY 40506, USA; zhengqiu.zhou@uky.edu (Z.Z.); e.ibekwe@uky.edu (E.I.); 2 Department of Molecular and Cellular Biochemistry, University of Kentucky, Lexington, KY 40536, USA

**Keywords:** mitochondria, oncometabolites, cancer, metabolic re-programming, aerobic glycolysis, mitochondrial dynamics

## Abstract

The mitochondrion is an important organelle and provides energy for a plethora of intracellular reactions. Metabolic dysregulation has dire consequences for the cell, and alteration in metabolism has been identified in multiple disease states—cancer being one. Otto Warburg demonstrated that cancer cells, in the presence of oxygen, undergo glycolysis by reprogramming their metabolism—termed “aerobic glycolysis”. Alterations in metabolism enable cancer cells to gain a growth advantage by obtaining precursors for macromolecule biosynthesis, such as nucleic acids and lipids. To date, several molecules, termed “oncometabolites”, have been identified to be elevated in cancer cells and arise from mutations in nuclear encoded mitochondrial enzymes. Furthermore, there is evidence that oncometabolites can affect mitochondrial dynamics. It is believed that oncometabolites can assist in reprogramming enzymatic pathways and providing cancer cells with selective advantages. In this review, we will touch upon the effects of normal and aberrant mitochondrial metabolism in normal and cancer cells, the advantages of metabolic reprogramming, effects of oncometabolites on metabolism and mitochondrial dynamics and therapies aimed at targeting oncometabolites and metabolic aberrations.

## 1. Aerobic Glycolysis in Normal and Cancer Cells

**Figure 1:**
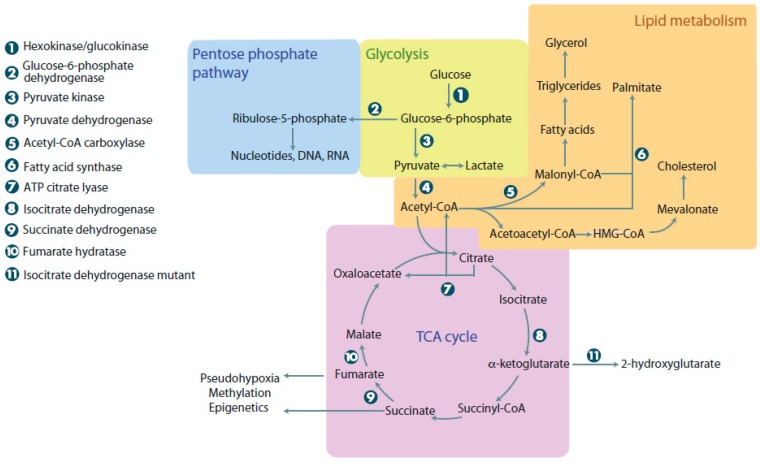
Metabolic pathways in normal and cancer cells. When glucose enters cells, it undergoes glycolysis, linking it to the pentose phosphate pathway, lipid metabolism, and the tricarboxylic acid (TCA) cycle. The pentose phosphate pathway can be used to make nucleotides, DNA, and RNA. Lipid metabolism is used for energy and synthesis of membrane components. The TCA cycle provides cells with intermediates for the electron transport chain and links many other metabolic reactions that occur in the cell. Cancer cells with mutations in metabolic enzymes have increased levels of 2-hydroxyglutarate, succinate, and malate, resulting in adverse cellular outcomes. ATP: Adenosine triphosphate.

Glucose is a vital molecule, providing energy for the cell and acting as a starting point for many reactions. Aerobic glycolysis begins when glucose enters the cell via insulin dependent or independent mechanisms, using the glucose transporter (GLUT) isoform membrane transporters [1]. In the cytoplasm, glucose undergoes glycolysis and generates pyruvate that is subsequently converted to acetyl-CoA by the pyruvate dehydrogenase (PDH) complex, located in the mitochondria. Acetyl-CoA will then act as a substrate for the first step of the tricarboxylic acid (TCA) cycle [2]. Mitochondria are biosynthetic and bioenergetic organelles, which function by utilizing molecules from the cytoplasm to facilitate the TCA cycle, urea cycle, ketogenesis, fatty acid oxidation, the electron transport chain, and synthetize important cellular macromolecules (amino acids, nucleotides, lipids, etc.) (Figure 1) [3]. Mitochondria contain a small 16 kb DNA genome, encoding tRNA, rRNA, and proteins essential for cellular respiration. Out of the 37 genes, 13 participate in the electron transport chain (Supplementary Table S1) [4]. Cells have hundreds of mitochondria, and typically, the number of mitochondria per cell is tissue-dependent. For instance, tissues like cardiomyocytes have more mitochondria than squamous epithelial cells [5]. Since each cell depends on mitochondria, diseases affecting this organelle have devastating consequences and affect multiple organ systems, like the nervous system, heart, and muscles [6]. As mitochondrial DNA is maternally-inherited, some mitochondrial diseases are caused by maternal inheritance of mutated mitochondria or loss-of-function (LOF) mutations in mitochondrial genes.

Cancer is theorized to be a somatic disease, arising from activating mutations in oncogenes and LOF mutations in tumor suppressor genes [7]. In contrast to the somatic mutation theory of cancer, Otto Warburg and others hypothesized that cancer is a metabolic disease, with respiratory insufficiency being the origin tumorigenesis [8,9,10]. 

While each theory provides a unique perspective on tumorigenesis, the reality is likely more complicated, and it may be that both mechanisms work in tandem. Geou-Yarh Liou et al., demonstrated, using in vitro and in vivo models of pancreatic adenocarcinoma, that K-Ras^G12D^ induced mitochondrial flux produces high levels of reactive oxygen species (ROS) that subsequently increase the activity of NF-κB1 and NF-κB2, leading to up regulation of epidermal growth factor receptor (EGFR) signaling [11]. In addition to K-Ras mutations, mitochondrial function is also influenced by the Phosphoinositide 3-kinase/Protein kinase B (PKB/AKT)/mechanistic target of rapamycin (PI3K/PKB(AKT)/mTOR) pathway. Makinoshima et al., demonstrated that levels of glucose 6-phosphate and 6-phosphogluconate were reduced in multiple EGFR-mutant lung adenocarcinoma cell lines, after inhibiting the PI3K pathway with Gedatolisib (PKI-587) [12]. Additionally, PI3K/mTOR inhibition suppressed membrane localization of GLUT1, resulting in down-regulation of aerobic glycolysis [12]. In contrast to the points above, some studizes demonstrated that the mitochondria could influence signaling cascades. The expression of mutated isocitrate dehydrogenase 1 and 2 (IDH1/2) or pre-treatment with D-2-hydroxyglutarate (D2HG) impaired the differentiation of erythroleukemia cells by inhibiting 5′-methylcytosine hydroxylase Tet methylcytosine dioxygenease 2 (TET2) [13]. Additionally, LOF mutations in succinate dehydrogenase (SDH) and fumarate hydratase (FH) have been found in paragangliomas and pheochromocytomas, and leiomas and renal cell cancer, respectively [14,15]. One way that high levels of succinate and fumarate may promote oncogenesis is through stabilization of Hypoxia-inducible factor 1-alpha (HIF1α) and inducing a pseudohypoxic state [16,17]. The role of the mitochondrion in tumorigenesis is complex, as it can be influenced by upstream signaling, feedback on that signaling, or generate its own signaling. 

## 2. Advantages of Metabolic Reprogramming in Cancer Cells

Normal mammalian cells rely on nutrients obtained from the extracellular environment to act as energy sources and precursors for macromolecule synthesis. Of these macromolecules, glucose can be argued to be the most important. Normal cells metabolize glucose into pyruvate that subsequently enters the TCA cycle to generate approximately 36 ATP in a process known as oxidative phosphorylation (Figure 1). The ATP generated is used to drive anabolic and catabolic reactions, vital for homeostasis and survival. Anabolic reactions involve synthesis of precursors for protein, nucleotide, and lipid synthesis. While non-proliferating cells rely on oxidative phosphorylation, rapidly proliferating cells are capable of fermenting glucose into pyruvate and then lactate, even with abundant oxygen availability, using a process termed aerobic glycolysis. There must be a benefit for rapidly dividing cells undergoing lactic fermentation in the presence of oxygen, even though it produces less ATP. The current most popular hypothesis suggests that rapidly proliferating cells, like tumor cells, use aerobic glycolysis to generate precursors for anabolic reactions synthesizing nucleic acids, lipids, and proteins [18].

### 2.1. Lipid Synthesis

To understand the benefit of aerobic glycolysis, a closer look needs to be taken at the precise role of the mitochondria in generating precursors. To do this, the mitochondria needs a continuous inflow of four carbon (4C) units to balance the outflow of 4C used for generation of amino acids, lipids, and nucleotides. The process of replenishing the TCA intermediates is termed anaplerosis. This process is vital for the uninterrupted turning of the TCA cycle and relies on the carboxylation of pyruvate [19,20,21]. In addition to 4C, the mitochondria also need 2C (two carbon) units that are derived from condensation reactions with acetyl-CoA. It is important to note that there are many sources for the derivation of 2C units. For example, fatty acids, pyruvate, acetate, and amino acids can all be used to generate 2C. Combining 4C and 2C units yields citrate, which can refuel the TCA cycle or exit the mitochondrion and be converted into oxaloacetate and acetyl-CoA, which will enter lipid synthesis.

Sources for cellular fatty acids (FAs) are exogenous (diet) or endogenous (anabolic biosynthesis). Homodimeric fatty acid synthase (FASN) is an enzyme responsible for catalyzing endogenous FA synthesis, using acetyl-CoA as a primer, malonyl-CoA as a 2C donor, and reduced nicotinamide adenine dinucleotide phosphate (NADPH) as a reducing agent. The major product of FASN is the 16C FA, palmitate [22,23,24]. In normal cells, FASN expression is present at low levels, as normal cells prefer to use circulating lipids for metabolic reactions, and dietary lipid intake is normally sufficient [25]. FASN is highly expressed in tissues with high levels of endogenous FA biosynthesis, like the liver, adipose tissue, and the breast [26]. Additionally, FASN expression is also found during embryogenesis, due to its role in producing lung surfactant, and the menstrual cycle as it i’s associated with endometrial cell proliferation [27,28,29]. 

In tumor cells, endogenous synthesis of FAs accounts for the majority of triacylglycerol FAs, and high levels of aerobic glycolysis provide energy and precursors for FA synthesis [30,31]. The increase in FA biosynthesis may be a response to high metabolic demand of cancer cells or an adaptation to reduced availability of serum-derived lipids. In cancer cells, it is necessary to maintain a constant production of lipids to supply the high rate of membrane production and lipid-based post-translational protein modifications [32,33]. It has been demonstrated that FASN is highly expressed in bone marrow samples of patients with multiple myeloma, as demonstrated by Wang and colleagues [34]. High levels of FASN expression were found in 22 out of 27 tumor samples, as determined by real-time polymerase chain reaction (RT-PCR), relative to normal tissue from healthy donors. Wang’s group also demonstrated that treating multiple myeloma cell lines highly expressing FASN with cerulenin, an inhibitor of FASN, inhibited its activity and induced apoptosis, suggesting that FASN can be a potential target in the treatment of multiple myeloma [34]. 

Several other key lipogenic enzymes can provide tumor cells with an endogenous source of FAs. ATP citrate lyase (ACLY) is an important enzyme involved in lipid biogenesis and is linked with glucose metabolism by catalyzing the conversion of citrate to oxaloacetic acid (OAA) and acetyl-CoA. High levels of ACLY expression and activity were found in lung, prostate, bladder, breast, liver, stomach, and colon cancers [30,35,36,37,38,39,40]. Interestingly, in non-small cell lung cancer, ACLY activity and over-expression was well correlated with stage, differentiation grade, and prognosis [35]. As ACLY is highly expressed in many tumor tissues, relative to healthy controls, it may be a potential drug target. Stable knockdown of ACLY in fetal liver clone 5.12 (FL5.12) cells resulted in impaired lipid synthesis, increased mitochondrial membrane potential, decreased cytokine-stimulated cell proliferation and impaired Akt-mediated tumorigenesis, in vivo [41]. Stable knockdown of ACLY induced differentiation of A549 lung adenocarcinoma and K562 chronic myelogenous leukemia cells, and suppressed tumor growth, in vivo. SB-204900, an ACLY inhibitor, induced permanent cell arrest in vitro and in vivo [42]. 

Another key lipogenic enzyme is acetyl-CoA carboxylase (ACACA), which is responsible for the rate limiting step of long-chain FA synthesis, because it carboxylates acetyl-CoA and produces malonyl-CoA [43]. ACACA has been shown to be over-expressed in advanced breast carcinomas and pre-neoplastic lesions that may progress to infiltrating breast carcinoma [44]. The ACACA gene was also found to be amplified in breast cancer and is associated with reduced survival [45]. Additionally, increased FA biogenesis may be associated with breast cancer in women with inherited germ line breast cancer 1, early onset (BRCA1) mutations. Recent studies found that breast-cancer-associated mutations in BRCA1 disrupt interaction between BRCA1 and phosphorylated inactivated ACACA (pACACA) [46]. Magnard et al., demonstrated that ACACA interacts with the BRCA1 C-Terminal (BRCT) domains of BRCA1 [46]. This interaction creates the BRCA1-pACACA complex, stabilizing inactive ACACA and reducing FA biogenesis. Mutations in the BRCT domain of BRCA1 (A1708E, P1749R, M1775R, R1835, Y1835) abolish this interaction. Therefore, BRCA1 with mutations affecting its BRCT domains, is unable to negatively regulate ACACA, leading to higher rates of FA synthesis. Also, it was demonstrated by Moreau et al., that BRCA1 knockdown significantly increased FA synthesis, providing evidence that BRCA1 can regulate ACACA activity, and that its tumor suppressor function may extend to regulating metabolism [47]. Additionally, the silencing of ACACA by RNAi in cell models of breast and prostate cancer impaired tumor cell proliferation and induced apoptosis, suggesting that ACACA is important for tumor cell survival [48,49]. Several small molecular inhibitors have been developed that target ACACA. Tofacitinib citrate (TOFA) was found to induce apoptosis in lung, colon, and breast cancer cell lines, and soraphen A induced apoptosis in prostate cancer cell lines [50,51,52]. These studies demonstrate the important roles of lipid biosynthesis in tumor metabolism. Further studies are needed to elucidate potential avenues for targeting these pathways, to improve patient cancer care and quality of life.

### 2.2. Nucleotide Synthesis

Rapidly proliferating cells require large amounts of purine and pyrimidine nucleotides for RNA and DNA synthesis. De novo nucleotide synthesis is complex and requires input from several pathways. The pentose phosphate pathway (PPP) provides the phosphoribosylamine backbone from ribose-5-phosphate (R5P), while glutamine is used as an amide donor [53]. Non-essential amino acids serve as precursors to purine and pyrimidine synthesis and methyl groups are obtained from the carbon (1C)/folate pool [54].

The Warburg effect provides cancer cells with a large influx of glycolytic intermediates, allowing tumor cells to shunt carbon from glycolysis into the PPP and synthesizing large amounts of R5P. Additionally the PPP also supplies large quantities of NADPH for detoxification of intracellular reactive oxygen species (ROS) [55]. Glucose-6-phosphate dehydrogenase (G6PD) catalyzes the first irreversible step of the PPP and generates NADPH. Due to the importance of this step, G6PD plays a critical role in regulating flux through the PPP, and high activity is expected in rapidly proliferating cells. 

One of the most commonly mutated genes in cancer cells is p53, and its loss results in the enhancement of both glycolytic and PPP flux [56,57]. p53 has been found to suppress glycolysis by increasing TP53-inducible glycolysis and apoptosis regulator (TIGAR) expression and lowering intracellular levels of fructose-2,6-bisphosphate (F26BP). F26BP, being an allosteric activator of phosphofructokinase-1 (PFK1), normally increases its activity. However, when p53 is mutated and TIGAR levels are low, F26BP increases PFK1 activity and drives glycolysis [57]. Additionally, it was found that wild-type p53 is capable of inhibiting PPP by complexing with G6PD and suppressing its activity. When p53 is mutated, it loses this ability and liberates G6PD [56]. As such, p53 mutations facilitate PPP flux, allowing cancer cells to generate precursors for nucleotide synthesis. In addition to p53, it has also been demonstrated that mutations in K-ras^G12D^ stimulate glycolysis and the non-oxidative PPP, in pancreatic ductal adenocarcinoma [58].

### 2.3. 1C Metabolism

Another important metabolic network is the mitochondrial metabolism of 1C units required for purine, pyrimidine, and methionine synthesis. Folate, an essential vitamin, carries 1C units in its activated tetrahydrofolate form (THF). 1C metabolism plays an important role in embryogenesis, growth, development, and is required for rapidly proliferating cells. It is well known that folate deficiency during pregnancy causes neural tube defects. While major pathways for the utilization of 1C units are cytosolic, the mitochondria also play an important role as they contain enzymes that generate THF-bound 1C units.

The mitochondrial pathway can generate 1C units using many donors, like glycine and betaine [59]. It was demonstrated that mitochondrial 1C enzymes, serine hydroxymethyltransferase (SHMT2) and methylene tetrahydrofolate dehydrogenase (MTHFD2), are highly over-expressed in cancer cells [60,61]. SHMT1 (cytosolic) and SHMT2 (mitochondrial) are methyltransferases that convert serine to glycine. During this reaction, a 1C unit is transferred from serine to THF-generating methylene-THF [62]. Since serine is a non-essential amino acid, it can be generated from the glycolytic intermediate, 3-phosphoglycerate (3-PG), further suggesting that high glycolytic flux during aerobic glycolysis produces important precursors for macromolecule synthesis [63]. SHMT2 expression has been found to be elevated in myc-transformed cells and human glioblastoma tumors and promotes cancer cell survival [64,65]. It has been found that inhibition of serine metabolism, by serine starvation or RNAi knockdown of SHMT2, caused accumulation of precursors and inhibition of cancer cell proliferation, by depleting 1C carbon units for purine biosynthesis [65,66,67]. MTHFD2 is an enzyme acting downstream of SHMT2 and is responsible for the conversion of methylene-THF to formyl-THF and methyl-THF for use in nucleotide synthesis and methionine recycling. MTHFD2 was found to be expressed in embryonic, tumor, and non-differentiated tissues [60,68]. During the conversion, nicotinamide adenine dinucleotide phosphate (NADP+) and nicotinamide adenine dinucleotide (NAD+) are used as cofactors, producing NADPH and reduced nicotinamide adenine dinucleotide NADH; this enables the production of reducing substances that can be used to protect the cell from ROS as well as provide reducing power for proline and fatty acid synthesis [67,69,70].

Alterations in mitochondrial metabolism are common in cancer. Furthermore, high aerobic glycolysis provides precursors used by cytosolic and mitochondrial pathways to generate FA and nucleotides. It will be of interest to explore targeting these pathways for cancer therapy.

## 3. Production of Oncometabolites and Effects on Normal Metabolism: 2-Hydroxyglutarate, Succinate, and Fumarate

Oncometabolite is a new term and refers to metabolites whose abundance is significantly increased in tumors relative to normal cells. There is now increasing evidence that oncometabolites are capable of contributing to the development and the progression of cancer. The current list of true oncometabolites is short and consists of 2-hydroxybutarate (2HG), succinate, and fumarate [71]. These oncometabolites are produced by mutations in the nuclear-encoded TCA enzymes, isocitrate dehydrogenase 1 and 2 (IDH1/2), succinate dehydrogenase (SDH), and fumarate hydratase (FH), and have been found in human cancers [72].

### 3.1. Mutations Affecting Isocitrate Dehydrogenase

There are three isoforms of IDH in human cells: IDH1 and IDH2 are homodimers that use NADP^+^ to catalyze the reversible conversion of isocitrate to alpha-ketoglutarate (α-KG) in the cytoplasm and the mitochondria, respectively. IDH3 is an NAD^+^-dependent heterotetramer that irreversibly decarboxylates isocitrate to α-KG in the TCA cycle [73]. While mutations have been observed in IDH1 and IDH2, IDH3 mutations have not been found in cancer. The most frequent mutations in IDH1 and IDH2 are associated with an arginine residue located in the catalytic sites, IDH1 (R132) and IDH2 (R172 or R140). These mutations have been found in human cancers, like low-grade glioma and glioblastoma, chondrosarcomas, cholangiocarcinomas, and acute myeloid leukemia (AML) [74,75,76,77,78,79,80,81].

**Figure 2:**
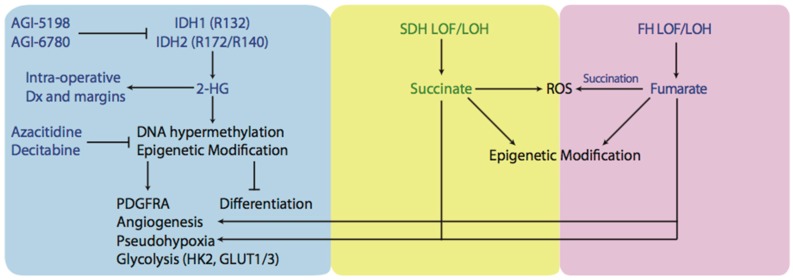
Schematic representation of an oncometabolic perspective of tumorigenesis. Mutated IDH1 and IDH2 produce oncometabolite known as 2-HG which goes on to exert several effects believed to trigger tumorigenesis. By inhibiting αKGDDs like cytosine hydroxylases, TET2, histone demethylases, IDH1/2 tumors exhibit CIMP phenotype as well as other epigenetic modifications and have inhibited differentiation. Tumours with high levels of 2-HG, succinate, or fumarate have been demonstarted to undergo a phenomenon known as pseudohypoxia and have increased levels angiogenesis. Furthermore, upregulation of HIF-1α target genes like HK2 and GLUT1 and GLUT3 were found in tumours containing IDH1/2 mutations and were associated with increased rates of glycolysis. Another potential cause of tumorigenesis is the production of ROS and tumours with SDH or FH LOF or LOH have been demonstrated to have high levels of ROS. Small molecular inhibitors have been designed to target IDH1/2 mutants (AGI-5198, AGI-6780) or reverse hypermethylation levels/CIMP phenotype (azacitidine, decitabine). Furthermore, 2-HG can be detected interaoperatively during tumour resection and be used as an indication of tumor margins. IDH: isocitrate dehydrogenase; 2-HG: 2-hydroxyglutarate; αKGDD: α-Ketoglutarate-dependent dioxygenases; TET2: Tet methylcytosine dioxygenase 2; CIMP: CpG island hypermethylator phenotype; HIF-1α: Hypoxia-inducible factor 1-alpha; ROS: reactive oxygen species; SDH: succinate dehydrogenase; FH: fumarate hydratase; LOF: loss of function; LOH: loss of heterozygosity.

Cancer-associated heterozygous mutations in IDH1 and IDH2 result in the formation of wild-type-mutant heterodimers with neomorphic activity, allowing the reduction of α-KG to 2-hydroxyglutarate (2HG) in the presence of NADPH [82,83,84,85]. This allows 2HG levels to accumulate to millimolar concentrations and exert effects on cell function (Figure 2). One example is the interference with the action of α-KG-dependent dioxygenases (αKGDD), like cytosine hydroxylases and histone demethylases [54,86,87]. Interestingly, while 2HG levels promote the hypermethylator phenotype in gliomas, known as the CpG island hypermethylator phenotype (CIMP), IDH1 mutations are associated with better overall survival [88,89,90,91]. Similarly, increased levels of DNA methylation were observed in AML samples with mutations in IDH1/2 or α-KG-dependent Tet methylcytosine dioxygenease 2 (TET2), suggesting that these mutations are mutually exclusive and could be part of a common pathway [92,93]. It has been shown that D2HG can inhibit TET enzymes and supports the notion that TET inhibition and DNA hypermethylation are important for IDH-driven tumorigenesis [94]. Flavahan and colleagues provided evidence for this hypothesis by demonstrating that DNA hypermethylation at CCCTC-binding factor (CFCF) binding sites results in reduced binding and insulator activity, causing aberrant enhancer activation of platelet-derived growth factor receptor-α (PDGFR) in gliomas [95]. The expression of IDH1/2 mutants in erythroleukemia cells is found to inhibit differentiation and facilitate a de-differentiation phenotype [13,87]. Similar findings were observed when IDH mutants were expressed in adipocyte cell lines and resulted in inhibition of differentiation [96]. These studies suggest that 2HG acts as an oncogenic driver by changing epigenetic programming and inducing dedifferentiation. 2HG has also been demonstrated to inhibit prolyl-hydroxylases (PDH), an αKGDD, resulting in a state known as pseudohypoxia. However, there have been contradictory results as to the effect of 2HG on PDH and induction of a pseudohypoxic state [97]. It was demonstrated that mice with IDH1 (R132H) mutation accumulated high levels of HIF-1α and upregulated HIF-1α target genes [98,99]. Overexpression of IDH1 R132H in human embryonic kidney cells 293 (HEK293T) and human primary glioblastoma cell line (U87MG) elevated HIF-1α levels [94,100]. However, this effect may be dependent on the enantiomer of 2HG, as l-2HG inhibits prolyl hydroxylase (PHD) protein activity while d-2HG promotes it, by acting as a PDH cofactor [97]. Inhibition of PDH by l-2HG increases HIF-1α and causes a shift towards glycolysis and angiogenesis [101,102]. By upregulating transcription of HIF-1α target genes, cancer cells increase the levels and activity of glycolytic enzymes, like hexokinase 2 (HK2) and glucose transporters (GLUT1 and GLUT3) [103]. This is important because HK2 is a major contributor to increased flux through glycolysis, and therefore, cells with high levels and activity of HK2 can undergo the Warburg phenotype.

Several new therapies are being developed to target IDH-mutant tumors. Some of them belong to the class of hypomethylating agents and others belong to direct IDH mutant enzyme inhibitors. Hypomethylating agents can be used in the context of the CIMP phenotype. Azacitidine and decitabine are two examples, and they function by inhibiting DNA methyltransferase (DNMT), and demonstrated clinical benefit in myelodysplastic syndromes (MSD) and AML [104,105,106]. In gliomas, the use of hypomethylating agents reduced DNA methylation of promoters involved in glial cell differentiation and reduced cell proliferation in human derived IDH1 mutant glioma xenografts [107,108]. IDH mutant inhibitors, like AGI-5198 and AGI-6780, have been tested preclinically and reduced 2HG levels, reversed histone and DNA hypermethylation, and promoted cell differentiation [109,110,111,112]. Some of these inhibitors are currently being evaluated in phase I clinical trials [113]. 

### 3.2. Mutations Affecting Succinate Dehydrogenase and Fumarate Hydratase

The accumulation of succinate and fumarate occurs in tumors with inactivating mutations in SDH and FH enzymes. Mutations in genes of various SDH subunits have been found in paragangliomas, pheochromocytomas, and gastrointestinal stromal tumors [86,114]. Studies have identified that SDH defects account for 10–30% of sporadic paragangliomas and 10–70% of familial paragangliomas [14]. Mutations in FH genes have been found in cutaneous and uterine leiomyomatas and renal cell cancer [15,115]. It has been found that SDH and FH mutations follow the hereditary pattern of tumor suppressor genes. Affected cells inherit a germline LOF mutation in one allele and the tumors that develop exhibit loss of heterozygosity (LOH) of the other wild-type allele, through somatic deletion or chromosomal loss [116]. 

There are several mechanisms that explain why LOF of SDH can lead to tumor formation (Figure 2). Similarly to 2 HG, high levels of succinate accumulate and leak out of the mitochondria via the dicarboxylate carrier and inhibit activity of HIFα PHDs in the cytosol, creating high levels of HIF1α and inducing a pseudohypoxic response [117]. To support this hypothesis, mutations in SDH subunits D and B in tumors from families with paragangliomas were found to activate the HIF1α pathway and be highly vascularized [118,119]. Furthermore, tumors deficient in FH also demonstrated high vascularization and significantly higher HIF1α levels as well as accumulation of fumarate and succinate [120]. The accumulation of both fumarate and succinate in FH-mutated paragangliomas makes sense, as FH catalyzes the subsequent step in TCA (Figure 1).

Other than creating a pseudohypoxic environment, SDH also participates in tumorigenesis by increasing ROS levels. SDH, in addition to its role in the TCA cycle, functions as complex II of the electron transport chain (ETC) and catalyzes oxidation of succinate to fumarate, generating reduced flavin adenine dinucleotide (FADH_2_), and donating its electrons to the ETC [71]. Using hepatoblastoma 3G (Hep3G) or hepatocellular carcinoma 3B (Hep3B) cells, lung carcinoma (A549) cells, and osteosarcoma cells, Guzy and colleagues demonstrated that RNA knockdown and pharmacologic inhibition of SDH-B, but not SDH-A, produced increased levels of ROS, HIF activation, and tumorigenesis [121]. Additionally, a transgenic mouse cell line (SDHC E69), overexpressing the mutated SDHC gene, was shown to overproduce the superoxide anion (O_2_^−^) and undergo higher rates of apoptosis. The clones that escaped apoptosis had an elevated number of DNA mutations and underwent transformation, as evidenced by the fact that cells formed benign tumors when injected into nude mice [122]. 

The loss of FH in renal cancer and leiomyomatosis leads to the accumulation of fumarate, and similarly to SDH loss, induces a pseudohypoxic state and accumulation of ROS [123]. To characterize FH mutations better, a database has been developed [124]. The mechanism of ROS production is different to SDH mutations. Succination is an irreversible, post-translational chemical modification of proteins formed by an addition reaction between fumarate and thiol residues [125]. High levels of fumarate can succinate glutathione, an intracellular antioxidant molecule, and reduce its antioxidant capacity [126]. NADPH, being a reducing equivalent, used in ROS detoxification, is used to metabolize the succinated glutathione. Therefore, succination of glutathione consumes NADPH and reduces the antioxidant capacity of the cell, increasing oxidative stress. Recently, it was demonstrated that succination of mitochondrial aconitase (ACO2) impairs its activity and succination of Kelch-Like ECH-associated protein-1 (KEAP1) inhibits its negative modulatory effect on nuclear factor-like 2 (NRF2) [127,128,129]. While NRF2 activation was previously demonstrated as a pro-tumorigenic event, its role in FH-deficient tumors remains unclear [130].

In addition to inducing a pseudohypoxic state and generating ROS, succinate and fumarate can also affect the epigenetic profile of the cell. Succinate and fumarate do this by inhibiting other αKGDD, like the Jumonji-C histone demethylases and the TET-family of 5-methylcytosine hydroxylases [14,131]. It was shown that knockdown of FH and succinate dehydrogenase complex, subunit A and B (SDHA/B) resulted in a significant increase in methylation on histone H3 Lysine 4 (H3K4), histone H3 Lysine 9 (H3K9), and histone H3 Lysine 79 (H3K79) [131]. Another study, using a human hepatocellular carcinoma G2 (HepG2) cells demonstrated that high levels of fumarate and succinate induced a loss in cell viability, activation of caspase 3/7, DNA fragmentation, and significantly increased levels of DNA hypermethylation [132]. 

## 4. The Link between Oncometabolites and Mitochondrial Dynamics

Metabolic derangements in cancer cells can result in increased rates of aerobic glycolysis and oxidative respiration. The production of oncometabolites has been shown to increase the invasive properties of cancer cells and facilitate epithelial to mesenchymal transition (EMT). Some studies have suggested a link between oncometabolites acting as paracrine signaling molecules that modulate tumor-infiltrating properties and the inflammatory response [133]. Several studies demonstrated that certain oncometabolites, like fumarate and D2HG, directly induce tumor cell migration and metastasis [134,135]. In addition to this, mitochondrial fragmentation has been found in invasive and metastatic tumor cells [136,137,138,139]. 

Mitochondrial fragmentation was previously demonstrated to be a property correlated with cell migration. It was found that mitochondria fragment and localize at the posterior regions of T cells, during chemotaxis. This is hypothesized to enable the mitochondria to provide energy, to facilitate migration [140,141]. Investigation into the role of mitochondrial dynamics and its role in tumorigenesis has been receiving more attention. The actions of mitochondrial-shaping proteins (OPA1, MFN1, MFN2, DRP1, FIS1) play a vital role in shaping and modifying the structure of mitochondria during fusion and division of this organelle [142]. For example, mitochondrial fragmentation has been observed during cell division in a cyclin-dependent kinase 1/dynamin-related protein 1 (CDK1/DRP1)-dependent manner in HeLa cells [143]. Another observation links mitochondrial dynamics to glycolysis, demonstrating that highly glycolytic cells have increased levels of mitochondrial fragmentation [144]. 

It is hypothesized that tumor metastasis is under metabolic control. This is supported by the finding that the transfer of mtDNA from highly- to weakly-metastatic cells increased their metastatic potential in mice [145]. While the exact mechanism for a mitochondrial role in metastasis is not yet clear, several studies shed light on this topic. Porporato et al., demonstrated that ETC overload and partial ETC inhibition promotes superoxide-dependent tumor cell migration and invasion [136]. Ferreira-da Silva et al., evaluated the expression of mitochondrial fusion and fission proteins in a panel of thyroid tumors and found that Drp1 overexpression was associated with migration and invasion [137]. Additionally, Drp1 expression was elevated in human invasive breast carcinoma cells that metastasized to regional lymph nodes and had higher levels of mitochondrial fragmentation, relative to non-metastatic breast cancer cells [138]. Using the human glioblastoma 251 (U251) cell model, Wan et al., demonstrated that hypoxic conditions increased Drp1 expression and facilitated mitochondrial fission, fragmentation, and tumor cell migration [139]. 

There is evidence for role of mitochondrial fusion and fission proteins in cell migration, invasion, and metastasis. However, there is a gap in knowledge linking oncometabolites to mitochondrial dynamics and their functional relationship with mitochondrial-shaping proteins. This represents a promising area for further study. 

## 5. Novel Therapies Targeting Metabolic Aberrations in Cancer Cells

The increased understanding of molecular pathways underlying cancer progression in tumors with mutations in the TCA cycle and macromolecular synthesis enzymes has opened the door to new therapies targeted at inhibiting these pathways. 

Alkylating agents, like temozolamide (TMZ), were approved in 2005 by the US Food and Drug Administration (FDA) for treatment of adult glioblastoma multiformes, along with ionizing radiation. However, tumors that express methylguanine methyltransferase (MGMT) are resistant to TMZ. Loss of MGMT expression confers sensitivity to TMZ and is often observed in tumors that have hypermethylation of the MGMT promoter. Tumors with the CIMP phenotype, previously described in this review, have been found to have higher levels of MGMT promoter hypermethylation and are more sensitive to TMZ. This can explain why IDH1/2 mutated glioblastomas and SDHB mutated paraganglioma and pheochoromocytoma are more sensitive to TMZ regiments and are associated with better prognoses [146,147]. 

As previously mentioned, oncometabolites may exert some of their oncogenic action by inhibiting αKGDDs. Therefore, reversing this dysfunction may be a promising therapeutic approach to cancers harboring these mutations. Several studies have shown that providing α-KG in excess can overcome the competitive inhibition of oncometabolites. High levels of α-KG can reverse pseudohypoxia and reduce the accumulation of 5mC, in vitro. Furthermore, α-KG demonstrated anti-tumor and anti-angiogenic properties in xenograft models by inducing PDH enzymes [148,149,150].

Another approach used for targeting oncometabolite pathways is to directly inhibit IDH1/2 mutants. Targeting these mutations is unique because small molecular inhibitors can inhibit IDH proteins and reduce 2-HG production. This approach has already been demonstrated and it was shown that the effects of 2-HG were reversible [13]. Additionally, Mazor et al. showed that the loss of mutant IDH1 does not completely remove the CIMP phenotype, indicating that mutant IDH1 and 2-HG may not be required for clonal expansion of glioma [151]. Inhibition of IDH2 (R140G) induced differentiation of AML cells, and inhibition of IDH1 (R132H) induced differentiation and impaired growth of glioma in in vitro and in vivo models [109,152]. Another approach to taking advantage of the IDH mutation is using is using 2-HG as a marker of tumour margins during intraoperative resection. It has been recently demonstrated that desorption electrospray ionization mass spectroscopy can detect 2-HG from tissue sections of surgically resected gliomas, intraoperatively. Levels of 2-HG correlated with tumor margins and could be mapped to 3D magnetic resonance imaging (MRI) reconstructions of tumors, allowing better localization of tumors (Figure 2). This novel approach could allow rapid molecular characterization of tissue samples during surgery and avoid the complex or time-consuming preparations associated with frozen sections [153].

## 6. Conclusions

Cancer cells can produce oncometabolites and alter normal cellular metabolic dynamics. Mutations in nuclear encoded mitochondrial enzymes can result in the production of oncometabolites, causing downstream effects. Another way that oncometabolites can be elevated is through increased flux through the TCA cycle [154]. The mitochondria are critical for oncometabolite production as well as for the increased rates of lipid and nucleotide synthesis observed in cancer cells. Two of the most studied effects of oncometabolites on cell function involve induction of a pseudohypoxic state and epigenetic regulation. However, more enzymes and pathways are likely involved and are functionally affected by oncometabolites that have not been elucidated yet. There is a growing link between oncometabolites and mitochondrial dynamics, and this is a potential exciting area of future research. Going forward, it will be important to answer the question of which metabolic pathways are drivers of cellular transformation and which are indirectly affected—passenger effects. As we understand more about the metabolic aberrations in cancer cells, due to the effect of mitochondria and oncometabolites on cancer metabolism, newer therapeutic strategies can be developed to improve cancer treatment.

## Supplementary Materials

The following are available online at www.mdpi.com/2076-3921/7/1/16/s1, Table S1: Mitochondrial Genes Encoding Respiratory Chain Components.
