# Supplementary material for: Metabolic Alterations in Cancer Cells and the Emerging Role of Oncometabolites as Drivers of Neoplastic Change
Source: Antioxidants (Basel). 2018 Jan 17;7(1):16. doi: 10.3390/antiox7010016 (PMC5789326; doi:10.3390/antiox7010016)
Supplement: Supplementary file 1 [file antioxidants-07-00016-s001.pdf]

mtDNA Gene	Function
MT-ND1	NADH-ubiquinone oxidoreductase chain 1 - this protein is a subunit of NADH dehydrogenase. Specifically, it is a subunit of respiratory chain Complex 1 belonging to a complex of proteins necessary to initiate NADH dehydrogenation and electron transfer to ubiquinone.
MT-ND2	Same function as MT-ND1
MT-ND3	Same function as MT-ND1
MT-ND4L	Same function as MT-ND1
MT-ND4	Same function as MT-ND1
MT-ND5	Same function as MT-ND1
MT-ND6	Same function as MT-ND1
MT-CYB	Encodes protein Cytochrome b which is a subunit of Ubiquinone Cytochrome c Reductase (Complex III) respiratory chain.
MT-CO1, MT-CO2, MT-CO3	Encodes proteins subunits Cytochrome c oxidases, which make up the Cytochrome c oxidase of respiratory complex IV.
MT-ATP6 MT-ATP8	Encodes protein ATP Synthase which is part of Respiratory complex V.

Supplementary Table 1. Mitochondrial Genes Encoding Respiratory Chain Components. Table lists mitochondrial genes (MD-ND1-6, MT-CYB, MT-COL1-3, MT-ATP6-8) with brief description of their function.
